# From a Pinched Nerve to a Fatal Prognosis: Sporadic Creutzfeldt-Jakob Disease Masquerading as Cervical Myelopathy

**DOI:** 10.7759/cureus.114217

**Published:** 2026-08-09

**Authors:** Julian Bayati

**Affiliations:** 1 Internal Medicine, Henry Mayo Newhall Hospital, Santa Clarita, USA

**Keywords:** acute encephalitis, csf protein 14-3-3, human prion disease, myoclonus, rapidly progressive dementia, rare case report, rare neurodegenerative disease, rt-quic, sporadic creutzfeldt-jakob disease (scjd), tau- protein

## Abstract

Creutzfeldt-Jakob disease (CJD) is a rare, rapidly progressive, and invariably fatal prion disease whose early clinical features often overlap with more common neurological conditions, leading to diagnostic delay. The following case describes a 53-year-old man with a history of hyperlipidemia and hypothyroidism who presented with a two-day history of right-sided facial and extremity numbness, right lower extremity weakness, and myoclonus of the right upper extremity. Initial stroke workup, including computed tomography (CT) and magnetic resonance imaging (MRI) of the brain, was unremarkable. Cervical spine imaging revealed moderate-to-severe stenosis at C5-C6, raising concern for a structural etiology. Neurosurgical evaluation determined that the degree of cervical stenosis was unlikely to fully account for the patient's diffuse neurological symptoms. Nevertheless, the patient was discharged on corticosteroids with plans for close outpatient follow-up and further evaluation. Approximately two weeks later, he returned with progressive right-sided weakness, spasticity, dysarthria, and new-onset cognitive decline. Repeat neuroimaging remained unremarkable, and electroencephalography demonstrated nonspecific diffuse slowing. The patient subsequently underwent cervical decompression surgery without clinical improvement. His condition rapidly deteriorated, with worsening encephalopathy, prominent myoclonus, and complete loss of speech. Cerebrospinal fluid (CSF) analysis subsequently revealed positive real-time quaking-induced conversion (RT-QuIC), markedly elevated tau protein (>20,000 pg/mL), and elevated 14-3-3 protein, establishing a diagnosis of probable sporadic CJD. The patient continued to decline and expired following transition to comfort care. This case highlights the diagnostic challenge posed by CJD when confounding structural abnormalities are present and underscores the importance of early consideration of prion disease in the setting of rapidly progressive neurological decline with cognitive impairment.

## Introduction

Prion diseases are a group of rare, fatal neurodegenerative disorders with a reported incidence of approximately 1-2 cases per million per year [1,2] and an incubation period ranging from 1 to 42 years [3]. The term "prion" is derived from "proteinaceous infectious particle" [2]. These diseases are characterized by the accumulation of abnormal, disease-specific protein aggregates in the brain. The central pathophysiological mechanism involves the conversion of the normal cellular prion protein (PrPC) to the pathological isoform (PrPSc), which leads to progressive neuronal death [1,4].

Human prion diseases are classified by etiology into sporadic (idiopathic), genetic, and acquired forms [1,3,5]. Sporadic forms include sporadic Creutzfeldt-Jakob disease (sCJD), which is the most common, accounting for approximately 85% of all CJD cases [1,3], as well as sporadic fatal insomnia (sFI) and variably protease-sensitive prionopathy (VPSPr) [1,2]. Genetic prion diseases result from mutations in the prion protein gene (PRNP) and include familial CJD, Gerstmann-Sträussler-Scheinker syndrome (GSS), and fatal familial insomnia (FFI) [1-3]. Acquired prion diseases include kuru (associated with ritualistic cannibalism), iatrogenic CJD (transmitted through medical or surgical procedures), and variant CJD (vCJD), which is linked to consumption of food contaminated with bovine spongiform encephalopathy-infected cattle products [1,3].

The initial symptoms of sCJD are often nonspecific, including headache, malaise, dizziness, and changes in personality, mood, or memory [5]. The clinical presentation may mimic stroke, neuropathy, other forms of dementia, movement disorders, or encephalitis [3].

Most patients eventually develop balance and gait disturbances, cognitive decline, and myoclonus [1,4], progressing to rapidly progressive dementia with extrapyramidal symptoms [3]. The disease is invariably fatal, with survival of less than 1 year in approximately 90% of patients [3-5]. At present, no effective treatment for prion disease exists [1,4].

Diagnosis of sCJD is challenging due to its variable and nonspecific clinical presentation and rapid progression [1,4]. Electroencephalography (EEG) may demonstrate characteristic periodic sharp wave complexes, particularly in later stages; however, its role has become complementary to more specific imaging and CSF biomarker tests [1,5].

Magnetic resonance imaging (MRI) has emerged as a highly sensitive diagnostic tool, with most centers reporting a sensitivity exceeding 90% for sCJD. Classical abnormalities include hyperintensity signals on diffusion-weighted imaging (DWI) or fluid-attenuated inversion recovery (FLAIR) sequences in the caudate nucleus, putamen, cerebral cortex, and/or thalamus [1,5]. CSF analysis may reveal elevated biomarkers of rapid neuronal injury, including 14-3-3 protein and phosphorylated tau [1]. The real-time quaking-induced conversion (RT-QuIC) assay has become a pivotal diagnostic tool, demonstrating high specificity (99-100%) and sensitivity (73-97%) for the diagnosis of sCJD [4].

The following case describes a 53-year-old man initially evaluated for focal neurological deficits and suspected structural spinal pathology. The patient rapidly progressed to severe cognitive and motor decline and was ultimately diagnosed with sCJD. Coexisting cervical spinal stenosis, concordant with his motor findings and surgically treatable, provided a plausible competing explanation that sustained diagnostic anchoring through an ultimately non-beneficial decompression. This case emphasizes the diagnostic complexity of rapidly progressive neurocognitive syndromes and the value of integrating clinical, imaging, EEG, and CSF biomarker findings for early recognition of prion disease.

This case emphasizes the diagnostic complexity of rapidly progressive neurocognitive syndromes and the value of integrating clinical, imaging, EEG, and CSF biomarker findings for early recognition of prion disease.

## Case presentation

A 53-year-old man with a 3-year history of hypothyroidism, managed with levothyroxine 25 mcg daily, and hyperlipidemia, managed with dietary modification, with no prior surgical history and no history of tobacco, alcohol, or recreational drug use, presented to the hospital with a 2-day history of right-sided facial and extremity numbness, right lower extremity weakness, and involuntary jerking movements of the right upper extremity. He was admitted for stroke workup.

First admission

On examination, vital signs were stable with a blood pressure of 135/85 mmHg, temperature 98.1 °F, oxygen saturation of 98% on room air, and heart rate of 72 beats per minute. General physical examination was unremarkable. Neurological examination revealed decreased sensation in the right upper and lower extremities, right hand spasticity, and right lower extremity strength of 4/5 with limited voluntary plantar flexion. The remaining extremities demonstrated full strength. Deep tendon reflexes were 2+ throughout, with no clonus or Babinski sign. Cranial nerves I-XII were intact, and finger-to-nose and rapid alternating movements were preserved.

Laboratory workup is summarized in Table 1. Notable findings included an elevated thyroid-stimulating hormone (TSH) of 11.15 mIU/L with a normal free T4 of 1.2 ng/dL, and a mildly elevated creatinine of 1.4 mg/dL attributed to large muscle mass and possible creatine supplementation. The toxicology screen was positive only for benzodiazepines, consistent with intravenous lorazepam administered in the emergency department. Urinalysis was unremarkable. Thiamine (vitamin B1) was mildly low at 72 nmol/L, though this result was not available until the second admission. Antinuclear antibody, hepatitis panel, human immunodeficiency virus, rapid plasma reagin, and heavy metal levels (arsenic, mercury, lead) were all negative.

**Table 1:** Selected laboratory findings on first and second admission PT: Prothrombin Time; APTT: Activated Partial Thromboplastin Time; IgM: Immunoglobulin M; IgG: Immunoglobulin G

Laboratory Test	First Admission	Second Admission	Reference Range
Hematology			
White blood cells	6.9	7.4	4.5-11 k/ul
Hemoglobin	15.4	15.7	14-16 g/dl
Platelet	209	177	140-400 k/ul
Mean corpuscular volume	77 (Low)	78 (Low)	79-101 fL
PT	12.8	—	12.2-14.2 SECS
APTT	26	—	23-35 SECS
Comprehensive Metabolic Panel			
Sodium	141	139	136-145 mmol/L
Potassium	4.5	4	3.4-5.1 mmol/L
Chloride	104	99	98-107 mmol/L
Carbon dioxide	26	27	22-29 mmol/L
Glucose	86	104 (High)	70-100 mg/dl
Blood urea nitrogen	22 (High)	21 (High)	6-20 mg/dl
Creatinine	1.4 (High)	1.4 (High)	0.7-1.2 mg/dL
Calcium	9.7	9.8	8.6-10 mg/dl
Magnesium	2	2.1	1.6-2.6 mg/dl
Total bilirubin	0.5	0.9	0-1.2 mg/dl
Aspartate aminotransferase	23	28	< 41 U/L
Alanine aminotransferase	19	23	< 42 U/L
Alkaline phosphatase	53	46	40-130 U/L
Total protein	6.6	7.3	6.6-8.7 g/dl
Albumin	4	4.3	3.5-5.2 g/dl
Globulin	2.6	3	2.3-3.5 g/dl
Lipid Panel			
Total cholesterol	250 (High)	—	< 200 mg/dL
Low-density lipoprotein	157 (High)	—	< 100 mg/dL
High-density lipoprotein	69	—	> 40 mg/dl
Triglycerides	120	146	< 150 mg/dL
Endocrine			
Thyroid-stimulating hormone	11.15 (High)	3.52	0.27-4.2 uIU/mL
Free T4	1.2	—	0.8-1.8 ng/dL
Hemoglobin A1c	5.50%	—	< 5.7%
Morning cortisol	—	12.41	6.02-18.4 ug/dL
Prolactin	—	15	2-18 ng/mL
Nutritional/Metabolic			
Vitamin B1 (Thiamine)	72 nmol/L (Low)	—	78-185 nmol/L
Vitamin B12	1034	—	232-1245 pg/mL
Folate	7.56	—	4.78-24.2 ng/mL
Copper	—	232	70-175 mcg/dL
Ceruloplasmin	—	18	14-30 mg/dL
Inflammatory Markers			
C-reactive protein (CRP)	0.3	—	< 4.1 mg/dL
Erythrocyte sedimentation rate (ESR)	8	—	0-22 mm/hr
Autoimmune/Infectious			
Antinuclear antibody	Negative		Negative
Human immunodeficiency virus	Negative		Negative
Rapid plasma reagin	Negative		Negative
Acute hepatitis panel	Negative		Negative
Tuberculosis test (Quantiferon)	—	Negative	Negative
Brucella IgM and IgG antibodies	—	Negative	Negative
Coccidioides IgM and IgG antibodies	—	Negative	Negative
Toxicology			
Urine toxicology screen	Benzodiazepines only	—	Negative
Ammonia	—	23	16-60 U/dL
Arsenic	< 3	—	< 23 mcg/L
Whole blood mercury	< 5	—	< 11 mcg/dL
Whole blood lead	1.3	—	< 3.5 mcg/dL

Computed tomography (CT) of the head and magnetic resonance imaging (MRI) of the brain were unremarkable. CT of the cervical spine demonstrated mild-to-moderate cervical spondylosis. MRI of the cervical spine revealed multilevel central spinal stenosis: mild at C3-C4, mild-to-moderate at C4-C5, moderately severe at C5-C6, and mild at C6-C7. Contrast-enhanced MRI of the cervical spine showed no evidence of myelitis or discitis. Carotid duplex ultrasonography demonstrated less than 50% stenosis bilaterally with minimal plaque. Electroencephalography (EEG) showed diffuse background slowing consistent with encephalopathy of metabolic, vascular, or degenerative origin.

Neurosurgery was consulted and noted that while moderately severe stenosis was present at C5-C6, the distribution of the patient's symptoms was more diffuse than expected from a single-level compressive lesion. Neurology, however, felt the cervical stenosis could account for the presentation, and anterior cervical discectomy and fusion (ACDF) at C5-C6 was initially planned. The patient felt the decision was premature and requested a second opinion. He was treated with methylprednisolone 80 mg intravenously every 8 hours for one day, transitioned to an oral prednisone taper, and discharged with plans for outpatient follow-up. Atorvastatin 40 mg daily was also initiated for hyperlipidemia at discharge.

Second admission

Approximately two weeks later, the patient returned with worsening of his prior symptoms and new-onset dysarthria and memory difficulties that were now affecting his activities of daily living. Vital signs were notable for a blood pressure of 137/106 mmHg. Neurological examination revealed gait abnormality secondary to right lower extremity weakness and spasticity, myoclonic jerks of the right upper and lower extremities, hyperreflexia of the right lower extremity with deep tendon reflexes of 4+, slurred speech, and expressive aphasia. The left side remained neurologically intact. Additional laboratory studies obtained during this admission are summarized in Table 1. TSH had normalized to 3.52 mIU/L.

Repeat CT of the brain was unremarkable. MRI of the brain without contrast, done on day 2 of admission, was read as unremarkable by radiology. A repeat MRI of the brain with and without contrast on day 4 of admission was also read as unremarkable. A third MRI of the brain with and without contrast on day 10 of admission was again interpreted as normal. All three MRI examinations included diffusion-weighted imaging (DWI) with corresponding apparent diffusion coefficient (ADC) maps. MRI of the cervical spine was unchanged. Three separate routine EEGs performed on different days during the hospitalization each demonstrated diffuse generalized slowing consistent with encephalopathy of metabolic, toxic, infectious, or neurodegenerative etiology, without epileptiform discharges.

On hospital day 1, intravenous thiamine 200 mg three times daily was initiated to empirically treat possible Wernicke encephalopathy as a contributor to the patient's encephalopathy; no clinical improvement was observed. On day 2, a lumbar puncture (LP) was recommended to evaluate for infectious, inflammatory, and neoplastic etiologies of the patient's progressive neurological decline, but the patient declined. Neurosurgery was reconsulted to reassess whether cervical stenosis could explain the progressive symptoms; however, cortical features, such as expressive aphasia and encephalopathy, are not typical of cervical myelopathy, which classically presents with upper motor neuron signs, gait difficulty, and hand clumsiness, raising concern that the patient's presentation could not be attributed to spinal cord compression alone. Prion disease was considered at this time, but remained low on the differential, and specific workup was not pursued. On day 3, neurology raised concern that one EEG pattern could represent seizure activity, and levetiracetam was started empirically but discontinued after three days without improvement.

By day 5, the rationale for cervical decompression was reassessed in the absence of any alternative structural explanation. The two brain MRIs obtained to that point had been read as normal, serial routine EEGs showed only nonspecific diffuse slowing, and CSF remained unavailable following the patient's refusal of lumbar puncture, leaving the C5-C6 stenosis as the only demonstrable structural abnormality identified. The patient had objective myelopathic signs referable to the cervical cord - right lower extremity weakness with spasticity, hyperreflexia (4+), and a spastic gait - concordant in level with the moderately severe C5-C6 stenosis on MRI. The working formulation was therefore one of possible dual pathology, with a potentially reversible compressive myelopathy accounting for the motor deficits while the cortical features remained unexplained and under investigation. Lumbar puncture was again discussed with the family; however, the decision was made to proceed with surgery. On day 6, ACDF at C5-C6 was performed. There was no postoperative neurological improvement.

On day 7, the patient became more confused with worsening aphasia and difficulty following commands. By day 8, myoclonus persisted, and the patient was noted to have episodes of staring into space. Speech had deteriorated to unintelligible mumbling. The family agreed to LP, and transfer to a tertiary care center was initiated for further evaluation of the altered mental status of unknown etiology. On day 9, the patient developed mild hypernatremia (sodium 152 mEq/L), attributed to poor oral intake, and was started on dextrose 5% in water.

LP was performed on day 10. Cerebrospinal fluid (CSF) analysis revealed a clear, colorless appearance with 1 white blood cell, 4 red blood cells, protein 39 mg/dL, and glucose 91 mg/dL. Oligoclonal bands were absent. CSF immunoglobulin G (IgG) index, myelin basic protein, Venereal Disease Research Laboratory (VDRL), coccidioides antibody, and West Nile virus testing were all negative. Prion-specific studies were not included in this initial sample; although prion disease had been raised on day 2, it remained low in the differential relative to more common treatable infectious, inflammatory, and neoplastic causes.

On day 12, the patient developed a fever. Blood cultures were obtained, and empiric antibiotics were initiated with vancomycin and cefepime. By day 13, myoclonus had progressed to bilateral involvement with weakness now affecting all extremities. The patient was no longer able to speak. Clinical suspicion for prion disease was raised by the primary medical team at this point, given the relentless decline despite all interventions; however, CJD-specific workup was not pursued at that time, as the patient remained pending transfer to a tertiary center for further specialized evaluation. Botulism was briefly considered in the differential, and antitoxin was administered without benefit.

On day 14, echocardiography was normal. Vancomycin was discontinued, and cefepime was transitioned to piperacillin-tazobactam due to concern for aspiration. The patient developed significant dysphagia, and peripheral parenteral nutrition was initiated. By day 15, mental status had further declined with increasing lethargy. All cultures returned negative. Tube feeding was started. The accepting neurologist at the tertiary care center contacted the team, reported that the case was highly suspicious for CJD, recommended pursuing prion-specific workup, and advised that transfer was no longer necessary. A family meeting was held to discuss the possibility of prion disease, and a second LP was performed with CSF sent for a prion panel and an autoimmune encephalitis panel.

Between days 16 and 18, the patient's neurological status markedly worsened, progressing to akinetic mutism. All infectious workup, including brucella serology, returned negative. The autoimmune encephalitis panel, including N-methyl-D-aspartate (NMDA) receptor antibodies, was negative. Comprehensive CSF viral studies, including herpes simplex virus and West Nile virus, were also negative. The patient developed deep vein thrombosis of the right lower extremity and was started on heparin. Goals-of-care discussions were held, and the family elected a do-not-resuscitate/do-not-intubate status.

**Figure 1:**
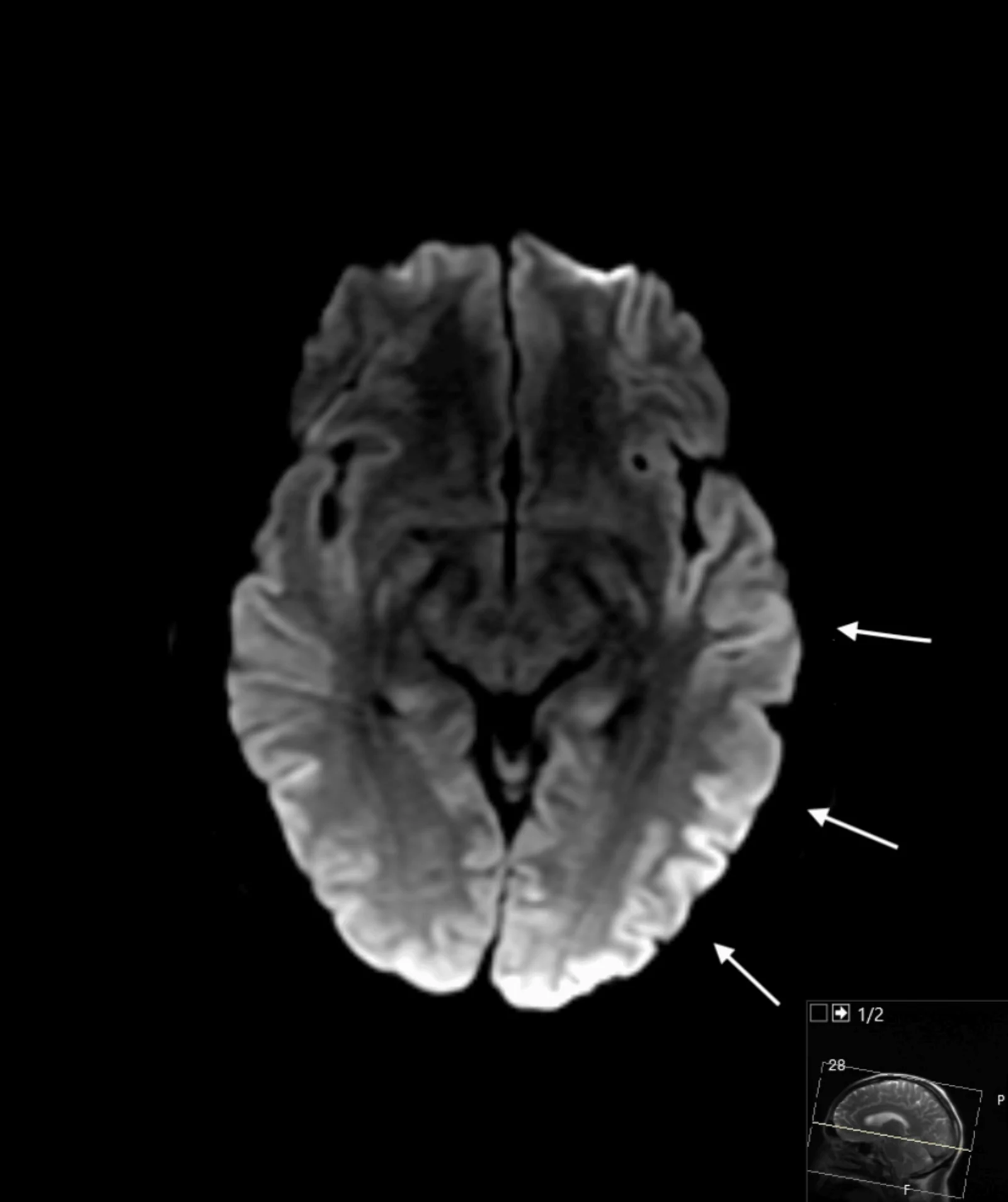
Axial diffusion-weighted imaging (DWI) of the brain demonstrating cortical ribboning with high signal intensity predominantly involving the left hemispheric cortex (arrows), consistent with Creutzfeldt-Jakob disease

On day 19, a second-opinion neurology consultation was obtained. Continuous EEG monitoring was obtained for the first time and demonstrated periodic sharp wave complexes, a pattern consistent with CJD. Targeted re-review of the prior brain MRI studies by the consulting neurologist, now guided by clinical suspicion for prion disease, identified left hemispheric cortical ribboning on DWI consistent with CJD that had not been appreciated prospectively (Figure 1).

On day 21, given the patient's history of hypothyroidism, empiric treatment for possible Hashimoto encephalopathy was initiated with pulse-dose intravenous methylprednisolone for five days while awaiting prion panel results. A slight initial improvement was noted, and on day 23, intravenous immunoglobulin (IVIG) was started for a 5-day course, overlapping with the steroid course. The steroid course was completed by day 25 and IVIG by day 27; however, no sustained meaningful clinical improvement was observed, and steroids were tapered and discontinued. Thyroid peroxidase (TPO) and thyroglobulin antibodies, which had been ordered concurrently, subsequently returned elevated; however, the lack of durable clinical response to steroids and IVIG argued against Hashimoto encephalopathy as the primary diagnosis. Notably, elevated thyroid antibodies are common in the general population and have poor specificity for Hashimoto encephalopathy, further limiting their diagnostic utility in this context.

On day 29, the CSF prion panel returned positive: real-time quaking-induced conversion (RT-QuIC) was positive, total tau protein was markedly elevated at greater than 20,000 pg/mL, and 14-3-3 protein was elevated at 157,702 AU/mL, confirming the diagnosis of probable sCJD. The family elected to transition to comfort measures. The patient passed away on day 31 of admission, approximately seven weeks after the onset of his initial symptoms.

The diagnostic and therapeutic timeline is summarized in Table 2.

**Table 2:** Timeline of clinical course, investigations, and interventions ACDF: anterior cervical discectomy and fusion; CJD: Creutzfeldt-Jakob disease; CT: computed tomography; DNR/DNI: do-not-resuscitate/do-not-intubate; DVT: deep vein thrombosis; DWI: diffusion-weighted imaging; EEG: electroencephalography; IVIG: intravenous immunoglobulin; LP: lumbar puncture; MRI: magnetic resonance imaging; RT-QuIC: real-time quaking-induced conversion

Timepoint	Events
First admission	Right-sided numbness, right lower extremity weakness, right upper extremity myoclonus; head CT and brain MRI reportedly normal; cervical MRI with moderately severe C5–C6 stenosis; EEG diffuse slowing. ACDF recommended despite neurosurgical concern that symptoms were more diffuse than the stenosis explained; deferred at the patient's request, discharged on steroid taper.
Interval (~2 weeks)	Progressive weakness with new dysarthria and memory difficulty.
Second admission	
Day 1	Myoclonus, hyperreflexia, expressive aphasia; empiric thiamine, no improvement.
Day 2	Brain MRI reported normal; lumbar puncture declined by the patient; prion disease was considered but ranked low.
Day 3	Empiric levetiracetam for possible seizure; no improvement.
Day 4	Repeat brain MRI with and without contrast was reported as unremarkable.
Day 5	C5–C6 stenosis was the only demonstrable structural abnormality; the decision was made to proceed with surgery.
Day 6	ACDF at C5–C6; no postoperative improvement.
Day 7-9	Worsening aphasia and confusion; speech reduced to mumbling; hypernatremia; the family agreed to LP.
Day 10	Third brain MRI reported normal; first lumbar puncture bland, infectious, and inflammatory studies negative; prion studies not sent.
Day 11-13	Bilateral myoclonus, loss of speech, botulism antitoxin given without benefit.
Day 14	Significant dysphagia, peripheral parenteral nutrition initiated.
Day 15	Outside neurologist advised high suspicion for CJD; second lumbar puncture sent for prion and autoimmune panels.
Day 16-18	Akinetic mutism; autoimmune, viral, and infectious studies negative; DVT treated with heparin; family elected DNR/DNI.
Day 19-20	Second-opinion neurology consult; continuous EEG showed periodic sharp wave complexes; targeted re-review of prior brain MRI identified left-hemispheric cortical ribboning on DWI (Figure 1).
Day 21-27	Pulse methylprednisolone then IVIG for possible Hashimoto encephalopathy; no sustained improvement.
Day 29-30	RT-QuIC positive, total tau >20,000 pg/mL, 14-3-3 157,702 AU/mL, establishing a diagnosis of probable sporadic CJD; transition to comfort measures.
Day 31	Death, approximately seven weeks after symptom onset.

## Discussion

This case illustrates the diagnostic challenge posed by sCJD when the initial presentation mimics more common and treatable neurological conditions. CJD is frequently misdiagnosed early in its course, with stroke, acute neuropathy, Hashimoto encephalopathy, and dementia among the most common initial diagnostic considerations [3,6]. In this patient, acute-onset right-sided focal deficits and myoclonus prompted an appropriate stroke workup, while the discovery of moderately severe cervical stenosis at C5-C6 introduced a plausible structural explanation that likely anchored the diagnostic evaluation. Despite neurosurgical assessment suggesting that the stenosis was insufficient to account for the diffuse neurological findings, cervical decompression was ultimately performed without clinical benefit. This sequence demonstrates how the presence of a plausible finding can lead to diagnostic anchoring - a cognitive bias in which early attachment to a working diagnosis delays consideration of alternative and less common etiologies. As the patient's symptoms progressed to include cortical features such as expressive aphasia, encephalopathy, and bilateral myoclonus, it became increasingly clear that the presentation could not be attributed to spinal cord compression alone.

MRI with diffusion-weighted imaging (DWI) is considered an essential investigation in suspected prion disease given its ready availability, short acquisition time, and high diagnostic accuracy, with reported sensitivity of up to 93-98% and specificity approaching 100%. Characteristic findings of sCJD on DWI and fluid-attenuated inversion recovery (FLAIR) sequences include high signal intensity in the cortex, caudate, putamen, or thalamus, or a combination of these regions, present in approximately 80% of cases [7,8]. DWI is more sensitive than FLAIR at detecting early cortical and subcortical changes, and restricted diffusion confirmed on apparent diffusion coefficient (ADC) mapping in more than one cortical region or the basal ganglia - in the absence of gadolinium enhancement and with sparing of white matter - points strongly toward a diagnosis of sCJD in the appropriate clinical context [7,9].

In this case, a brain MRI was obtained on multiple occasions during the second admission and was initially interpreted as unremarkable. After the neurologist at the tertiary care center raised clinical suspicion for CJD on day 15 and recommended prion-specific CSF testing, a second-opinion neurology consultation on day 19 prompted targeted re-review of the prior imaging, which identified left hemispheric cortical ribboning on DWI sequences consistent with prion disease. This sequence illustrates a well-recognized challenge: the subtle cortical DWI changes of early sCJD can be difficult to detect without a high index of clinical suspicion, and studies have shown that diagnostic sensitivity of MRI for CJD varies significantly depending on the interpreter's familiarity with prion disease neuroimaging [7,9]. This case reinforces the value of obtaining expert neuroimaging review when a rapidly progressive neurological decline does not conform to an established diagnosis.

Electroencephalography (EEG) plays an important but stage-dependent role in the diagnosis of sCJD. The spectrum of EEG findings evolves with disease progression: in the first clinical stage, characterized by dementia and focal symptoms, EEG typically shows only nonspecific changes such as generalized or focal delta-theta slowing and poor reactivity. Periodic sharp wave complexes (PSWCs) characteristically emerge during the second stage, when progressive dementia, widespread myoclonus, and pyramidal signs develop, and tend to simplify and broaden in the terminal third stage. The overall sensitivity of EEG for detecting PSWCs ranges from 40% to 70% across studies, and PSWCs are typically detected late in the disease course. Additionally, PSWCs are prominent during wakefulness and tend to disappear during sleep, and may be suppressed by external stimuli or sedative medications such as benzodiazepines - observations that can help differentiate CJD-associated PSWCs from periodic discharges of other etiologies [10].

In this case, three routine EEGs performed on separate days during the second admission demonstrated only diffuse generalized slowing, consistent with the expected findings in early-stage sCJD. It was not until day 19, when continuous EEG monitoring was obtained for the first time, that PSWCs were identified - a finding consistent with the patient's clinical progression to the second stage of disease. This sequence highlights that a normal or nonspecifically abnormal EEG does not exclude CJD, and supports the practice of obtaining serial and continuous EEG monitoring when clinical suspicion for prion disease persists despite initially nondiagnostic recordings [10].

The diagnostic evaluation in this case was further complicated by competing diagnostic hypotheses, most notably Hashimoto encephalopathy. Hashimoto encephalopathy, also referred to as steroid-responsive encephalopathy associated with autoimmune thyroiditis (SREAT), can present with features overlapping with CJD, including cognitive decline, myoclonus, tremor, seizures, and ataxia [11]. Given the patient's history of hypothyroidism, empiric treatment with pulse-dose corticosteroids was initiated for possible Hashimoto encephalopathy while awaiting thyroid antibody results. Slight initial improvement was observed, likely attributable to a transient reduction in neuroinflammation rather than treatment of the underlying disease [6]. However, the subsequent clinical course was characterized by relentless deterioration despite completion of both corticosteroids and IVIG, arguing against an immune-mediated etiology. Importantly, thyroid antibodies may be elevated in up to 10% of the general population, and their pathogenic role in Hashimoto encephalopathy remains unclear, limiting their diagnostic specificity [11]. Beyond Hashimoto encephalopathy, other treatable etiologies were also considered. The administration of botulism antitoxin on day 13 was prompted by a reported history of raw honey consumption. Although clinical suspicion for botulism was low, the decision reflected the team's commitment to pursuing all potentially treatable etiologies in the setting of diagnostic uncertainty. No clinical benefit was observed.

CSF analysis plays a central role in the diagnostic evaluation of suspected prion disease. CSF in CJD is characteristically bland; pleocytosis can occur but is rare, and the absence of inflammatory markers helps exclude infectious and autoimmune etiologies [5]. For over two decades, the principal CSF biomarkers have been proteins released during neuronal and glial injury, including 14-3-3 protein, tau, S100b, and neuron-specific enolase. While abnormalities of these markers are found in over 80% of CJD patients, they reflect rapid neural tissue damage rather than prion-specific pathology and may therefore be elevated in many mimic conditions [5,11].

The development of RT-QuIC represented a significant diagnostic advance. First described in 2010, this technique exploits the ability of pathological prion protein (PrPSc) to induce recombinant prion protein to misfold and form amyloid fibrils, a process monitored in real time through thioflavin T fluorescence with characteristic sigmoidal aggregation kinetics [9]. RT-QuIC has proven to be both highly sensitive (73-97%) and highly specific (99-100%) for diagnosing CJD and is now incorporated into standard diagnostic criteria [4,12].

In this case, the initial LP on day 10 revealed bland CSF with normal cell count, protein, and glucose, which was consistent with prion disease but also nonspecific. The second LP on day 15 included prion-specific testing, and the results returned on day 29 established a diagnosis of probable sporadic CJD: RT-QuIC was positive, total tau was markedly elevated at greater than 20,000 pg/mL, and 14-3-3 protein was elevated at 157,702 AU/mL. The delay in obtaining LP, initially due to patient refusal and subsequently due to prioritization of surgical intervention, contributed to the prolonged diagnostic timeline and underscores the importance of early CSF evaluation in any case of rapidly progressive neurological decline.

Although no effective treatment for prion disease currently exists, timely diagnosis remains critically important. Multiple therapeutic approaches have been investigated, including antiviral agents, such as acyclovir and interferon, amantadine, and various anticonvulsants for myoclonus and seizures, but none have demonstrated significant effects on disease course or survival [13]. Current research efforts are focused on novel strategies targeting PrPSc, including antisense oligonucleotide therapies directed at PRNP mRNA and a monoclonal antibody (PRN100) targeting the normal cellular prion protein (PrPC); however, human trials have not yet yielded effective treatments [1,4,13].

The value of early diagnosis lies not in curative intervention but in enabling appropriate goals-of-care discussions, avoiding unnecessary invasive procedures, and ensuring comprehensive palliative support. CJD has profound consequences for patients and their caregivers, who may experience significant emotional and psychological distress as the disease progresses rapidly. In the terminal stages, palliative care focuses on symptom relief, family support, and preservation of the patient's dignity [14]. Notably, despite progressing to near-complete akinetic mutism, this patient demonstrated preserved emotional awareness - on one occasion becoming tearful without vocalization upon recognizing his physician - underscoring the devastating nature of this disease and the importance of early, transparent communication with families regarding prognosis and goals of care.

This patient's seven-week disease course from symptom onset to death underscores the aggressive nature of sCJD. Clinicians should maintain a high index of suspicion for prion disease in any patient with rapidly progressive neurological decline, even when a plausible alternative diagnosis is present.

Lessons learned and diagnostic pitfalls

Several aspects of the diagnostic pathway could have been approached differently. The clues that should have prompted earlier suspicion for CJD were the cortical features: expressive aphasia, encephalopathy, and myoclonus, which are not explained by cervical cord compression, together with the pace of decline over weeks despite brain imaging reported as normal. These should have been treated as the dominant diagnostic problem rather than cervical stenosis, which, being both plausible and surgically actionable, likely anchored the evaluation toward a structural explanation and contributed to a decompression that produced no benefit. Lumbar puncture with prion-specific testing and targeted re-review of the brain MRI with DWI sequences should have preceded rather than followed cervical decompression. Continuous EEG might have identified periodic sharp wave complexes earlier, although these are stage-dependent, and earlier monitoring may still have been nondiagnostic. Although no disease-modifying therapy exists, earlier diagnosis would plausibly have avoided surgery and allowed goals-of-care discussions weeks sooner.

A limitation of this case is that neuropathological examination via brain biopsy or autopsy was not performed, which is required for a definitive diagnosis of sCJD. However, the combination of characteristic clinical features, progressive EEG changes, cortical ribboning on DWI, and positive CSF biomarkers, including RT-QuIC, supports a diagnosis of probable sporadic CJD per established diagnostic criteria [1].

## Conclusions

This case highlights the diagnostic complexity of sporadic Creutzfeldt-Jakob disease when the initial presentation mimics more common and treatable neurological conditions. The patient's focal right-sided deficits and myoclonus, combined with the finding of moderately severe cervical spinal stenosis at C5-C6, initially directed the clinical evaluation toward structural spinal pathology. Despite neurosurgical assessment suggesting that the stenosis was insufficient to explain the diffuse neurological findings, cervical decompression was ultimately performed without clinical benefit. This sequence illustrates the concept of diagnostic anchoring, in which the presence of a plausible structural abnormality can delay consideration of alternative and less common diagnoses. Given the patient's history of hypothyroidism, empiric treatment for possible Hashimoto encephalopathy was initiated with corticosteroids and intravenous immunoglobulin while CSF biomarker results for prion disease were pending. Thyroid antibodies subsequently returned elevated; however, the lack of clinical response to these therapies helped exclude immune-mediated etiologies.

The progression from isolated focal deficits to rapidly progressive cognitive decline, bilateral myoclonus, and akinetic mutism prompted CSF biomarker evaluation, which confirmed the diagnosis through a positive RT-QuIC assay. The delay in obtaining a lumbar puncture, initially due to patient refusal and subsequently due to prioritization of surgical intervention, contributed to a prolonged diagnostic timeline. This case underscores the importance of maintaining a high index of suspicion for CJD in any patient presenting with rapidly progressive neurological decline, even when plausible alternative diagnoses are present. Prompt recognition of prion disease allows for meaningful goals-of-care discussions with families and helps avoid unnecessary invasive procedures.
